# Techno-Economic Feasibility of Producing High-Protein Tofu from Chickpeas: Process Design and Nutrient Recovery

**DOI:** 10.3390/foods14183206

**Published:** 2025-09-15

**Authors:** Ossama Dimassi, Lina Jaber, Imad Toufeili, Krystel Ouaijan, Shady Hamadeh

**Affiliations:** 1 Department of Research, American University of Europe, New York, NY 10017, USA; 2 Environment and Sustainable Development Unit, Faculty of Agricultural and Food Sciences, American University of Beirut, Beirut P.O. Box 11-236, Lebanon; lj01@aub.edu.lb (L.J.); shamadeh@aub.edu.lb (S.H.); 3 Department of Agriculture, Faculty of Agricultural and Food Sciences, American University of Beirut, Beirut P.O. Box 11-236, Lebanon; 4 Department of Nutrition and Food Sciences, Faculty of Agricultural and Food Sciences, American University of Beirut, Beirut P.O. Box 11-236, Lebanon; toufeili@aub.edu.lb (I.T.); ko07@aub.edu.lb (K.O.)

**Keywords:** chickpea tofu, plant-based protein, sustainable food systems, protein retention, tofu production, techno-economic modeling, low allergenic risk, sustainable development goals, emergency food systems

## Abstract

This study presents a comprehensive assessment of tofu production from whole chickpeas as a plant-based protein alternative for sustainable food systems and humanitarian use. A novel process comprising soaking, wet milling, starch sedimentation, thermal coagulation, and optional drying yielded tofu with 56.2% protein (dry basis). Byproducts, including starch and okara, were also recovered and characterized. Nutrient recovery analysis, relative to seed nutrient content, showed that tofu retained most of the protein (59.1%) and fat (43.2%), okara accounted for the majority of fiber (34.5%) with residual protein (13.5%) and fat (16.7%), while the starch fraction primarily contained net carbohydrates (21.6%). Techno-economic modeling showed that fresh tofu can be produced with minimal inputs and an estimated thermal requirement of 0.798 kWh/kg, while tofu powder required 4.109 kWh/kg; both represent idealized values assuming no heat loss or system inefficiency. Theoretical energy minima were estimated under idealized assumptions, and broader environmental and food security implications are discussed as perspectives. Unlike soy, chickpeas carry a low allergenic risk, which may enhance suitability for population-wide feeding interventions. Broader implications for sustainable development goals (hunger, health, climate action) and humanitarian applications are discussed as perspectives. Chickpea tofu may represent a viable shelf-stable protein platform for local and emergency food systems.

## 1. Introduction

Meeting the protein requirements of a growing global population while remaining within environmental limits calls for a decisive pivot toward low-impact plant proteins. Recent cradle-to-grave life-cycle modeling shows that pulses release far less greenhouse gas per kilogram of protein than either livestock products or most cereals [1]. Pulses also supply concentrated protein and dietary fiber while demanding comparatively little land and water [2,3].

Soybean products currently dominate the plant-protein market, yet soy is among the foods most frequently implicated in serious immunoglobulin E (IgE)-mediated reactions. Heat-stable storage allergens, such as Gly m 5 and Gly m 6, persist even after standard processing [4]. Dependence on imported soy magnifies risk: customs data show that Lebanon imported about 40,448 t of whole soybeans in 2023, valued at roughly USD 27.8 million, with 86 % being sourced from Brazil [5].

Soy tofu typically contains 40–55% protein on a dry basis [6], while chickpea curds have shown comparable or higher firmness and retention linked to 11S globulins [7]. The growing regulatory acceptance of chickpea protein concentrates (≥60% protein; [8]) further underscores the relevance of exploring chickpea-based tofu.

Chickpea (*Cicer arietinum* L.) has emerged as the third-largest grain legume worldwide, reaching more than 16 million t in 2022 and sustaining a 2.5% compound annual growth rate (CAGR) since 2010 [9]. Chickpeas contain 17–24% protein, balanced essential amino acids, resistant starch, and appreciable iron, zinc, and folate [10,11]. Pournaki et al., 2024 [12] report a protein digestibility–corrected amino acid score (PDCAAS; a digestibility-adjusted measure of how well a protein meets human amino-acid requirements) of about 0.76 for chickpea protein, with digestibility shifting by cultivar and processing method. Updated surveys indicate high water- and oil-holding capacity (2.0–2.7 g g^−1^), 80–90% foaming stability, and gelation at 10–14% protein, with values that are similar to those for soy isolates [13]. Agronomic work lists drought-tolerant Desi and Kabuli cultivars capable of fixing atmospheric nitrogen and thriving under rain-fed conditions in more than fifty countries [14].

Despite growing attention to nutrition interventions, child malnutrition remains a persistent global challenge: stunting has declined only slightly (from 26.4 percent in 2012 to 23.2 percent in 2024), and wasting shows virtually no improvement (7.4 percent in 2012 versus 6.6 percent in 2024) [15]. Supplementary feeding remains the cornerstone of treatment for wasting, yet most products rely on imported peanut- and soy-based ingredients that carry allergenicity risks and long supply chains [16].

Locally processed pulse-based foods can improve nutrition security where animal protein is scarce or costly. For example, a Bangladesh program demonstrated that a chickpea-based supplement improved child growth while fostering domestic manufacturing capability [17]. In breadmaking, substituting 10% wheat flour with chickpea flour did not affect consumer acceptance while raising protein to 12.9% and lowering predicted glycemic load [18], underscoring the versatility of chickpea ingredients.

Previous studies on alternative legumes showed that chickpeas and fava beans yield firmer gels than lentils or mung beans due to their higher 11S globulin content [7]. Similarly, soy–chickpea blends enhanced fiber but reduced protein levels compared with pure soy tofu [19]. However, systematic data remain lacking on the yield efficiency, energy intensity, and scalability of chickpea-only tofu production, which this study directly addresses.

Despite these advantages, the development of tofu-style products from chickpeas remains underexplored, especially in terms of yield efficiency, protein retention, energy use, and cost under small-scale or low-resource conditions. While chickpea proteins show good gelation and nutritional value, their higher starch content introduces processing challenges that differ from soy-based systems.

This study addresses these gaps by evaluating the feasibility of producing tofu from whole chickpeas. It analyzes the nutritional transformation, economic viability, environmental impact, and food security implications of this process, with a specific focus on household-scale and humanitarian applications. Conventional soy tofu is used as a benchmark throughout.

## 2. Background

### 2.1. Nutritional Composition and Allergenicity

Compositional analysis show that chickpea protein quality approaches that of soy when paired with cereal staples, with chickpeas providing higher levels of resistant starch, dietary fiber, and several micronutrients [2,10]. Kumar et al. (2025) report polyphenol concentrations of 1.2–1.8 mg gallic acid equivalents g^−1^ and oligosaccharide profiles with prebiotic effects for chickpeas [11].

Chickpeas also exhibit markedly lower concentrations of major soy allergens [3,20], an advantage for broad-based or institutional feeding. The major chickpea protein fractions include globulin (53–60%), glutelin (19–25%), and albumin (8–12%), with an 11S:7S (legumin/vicilin) ratio of 4–6:1, with legumin contributing most of the sulfur-containing amino acids [12]. Cross-legume syntheses indicate similar indispensable amino acid scores across chickpea, soy, lupin, and lentil proteins [21,22], supporting chickpea’s nutritional viability in curd-based applications.

### 2.2. Functional and Technological Properties of Chickpea Proteins

Chickpea proteins possess functional properties conducive to curd formation, including favorable gelation behavior and water-holding capacity [9,23]. Protein concentrates demonstrate solubility above 60% at pH 7, emulsifying activity of 42–54 m^2^ g^−1^, least gelation concentration near 10%, and water absorption up to 2.5 g g^−1^. At 12% protein, chickpea gels exhibit a hardness of 7–10 N, yielding curds comparable in firmness to commercial soy tofu [13]. These gels form at calcium chloride concentrations similar to those used in soy processing. Chickpea proteins also exhibit substantially lower trypsin inhibitor activity than soy, which reduces the need for prolonged heat treatment and improves digestibility [3,4]. This further enhances their suitability for small-scale or resource-limited processing environments.

### 2.3. Conventional Tofu Production and Processing Constraints

Soy-based tofu production involves soaking, wet grinding, blanching, filtration, coagulation with calcium salts, and pressing. Protein unfolding and ionic bridging during heat-coagulation govern curd yield and texture [24]. Target values for soymilk include solids of 9–10% and a calcium-to-protein ratio around 30 mg g^−1^ [25].

However, the process is energy- and water-intensive, and 17–29 % of the seed protein remains in okara [26]. Increasing soymilk solids from ~7 to 9° Brix improves hardness but slightly lowers yield, while reducing set time increases cohesiveness [6]. Yet major allergens like Gly m 5 and Gly m 6 remain stable after heating [4], raising concerns for allergen-sensitive populations. Alternative coagulants such as MgCl_2_ and steep-water effluent modulate yield, nutrient recovery, and sensory outcomes [27]. While these principles provide a reference framework, adaptations are essential when applying them to chickpeas, given the compositional and functional differences.

### 2.4. Process Analogs and Applications of Chickpea-Based Tofu

Bench-scale trials confirm that chickpea milk can yield tofu-like curds with comparable texture, provided process conditions are adjusted. Partial replacement experiments, in which 10–30% of soy is substituted with chickpea flour, have been shown to reduce pH, increase curd hardness, and maintain acceptable protein levels [19]. Microenterprise models using “Soy Kit” equipment have successfully adapted this process to chickpeas, with capital costs under USD 100 and pay-back periods of less than six months [28]. At a larger scale, pilot-plant tests demonstrate that small-batch curd yields and textures correlate strongly with full-scale outcomes [7], while multi-location trials identify protein and stachyose content as positive predictors of yield and firmness, and oil and sucrose as negative predictors [29].

Beyond curd production, chickpea starches and proteins have found applications in gluten-free pasta and dairy-free formulations. Comparative reviews indicate that wet fractionation, as opposed to dry air classification, yields higher-purity protein and starch fractions suitable for novel food systems [12]. Additionally, dried okara, whether from chickpea or soy, has been successfully incorporated into gluten-free bread [30] and aquaculture feed formulations [31], illustrating opportunities for circular economy integration.

While these trials and applications confirm the technical feasibility of chickpea-based tofu, a systematic approach to process optimization, yield efficiency, and coproduct valorization remains lacking. This work attempts to address these process variables.

### 2.5. Low-Cost Systems and Humanitarian Relevance

Low-cost tofu production systems using basic heating and filtration units have shown promise in resource-limited settings. Soy Kit processors, costing under USD 100, allow one operator to convert 600 g of legumes into 3.5 L of hot milk in 45 min; further, over 80% of franchisees are women, with a median pay-back period of 4.9 months [28]. While tofu is not yet explicitly listed in WFP procurement guidelines, chickpea flour and other pulse-based nutritional commodities are prioritized, indicating procurement compatibility [32]. In Lebanon, FAO’s OCOP (One Country One Priority Product) roadmap aims to increase domestic chickpea production from 29% to 40% of national demand by 2030, with investments in quality and rural employment [33].

Field-based supplementation programs in Bangladesh using chickpea-based supplements met Codex quality standards and improved child growth [17]. Reviews highlight the potential of decentralized pulse processing to shorten supply chains, retain value locally, and enhance diet diversity [34]. Taken together, these findings suggest chickpea tofu microenterprises can serve as a scalable nutrition-responsive intervention.

### 2.6. Motivation for Process Adaptation

Despite their nutritional and functional promise, chickpeas contain substantially more starch than soybeans, which can interfere with protein coagulation and weaken curd structure. To address this, the present study introduced a starch sedimentation step before coagulation to increase protein concentration, enhance curd firmness, and improve overall process clarity. In parallel, heating and filtration steps were simplified to address the constraints of small-scale or resource-limited production environments. This approach supports the development of a low-input high-protein tofu system adapted to decentralized contexts. The following sections describe the experimental design used to evaluate the system’s performance in terms of yield, nutritional composition, techno-economic feasibility, and environmental impact.

## 3. Materials and Methods

### 3.1. Production of Chickpea Tofu

Desi-type chickpeas (*Cicer arietinum* L.) were purchased from a local wholesale market in Beirut/Lebanon. Before use, chickpeas were hand-cleaned to remove foreign material and visibly defective grains.

Chickpea tofu was produced in nine unit operations (Table 1). Chickpeas were soaked in three parts of water (1:3, *w*:*w*; 10 h, 22 °C), and drained. For slurry preparation, soaked chickpeas were blended with water at a 1:4 volume-to-volume ratio (soaked seeds/water). Based on measured moisture contents (47% for soaked seeds, 13.5% for raw seeds) and a soaked-seed density of 1.15 kg L^−1^ [35], the 1:4 *v*:*v* slurry corresponded to a raw chickpea-to-water ratio of 1:5.7 (*w*:*w*), consistent with values used in tofu studies [6,7].

After muslin filtration, the milk was boiled (100 °C, 5 min), cooled to 85 °C, and coagulated by anhydrous CaCl_2_ (2.5 g L^−1^). Curds were ladled into cloth-lined molds, gravity-drained for 60 min, and refrigerated overnight (4 °C). The operating parameters are presented in Table 1.

**Figure 1:**
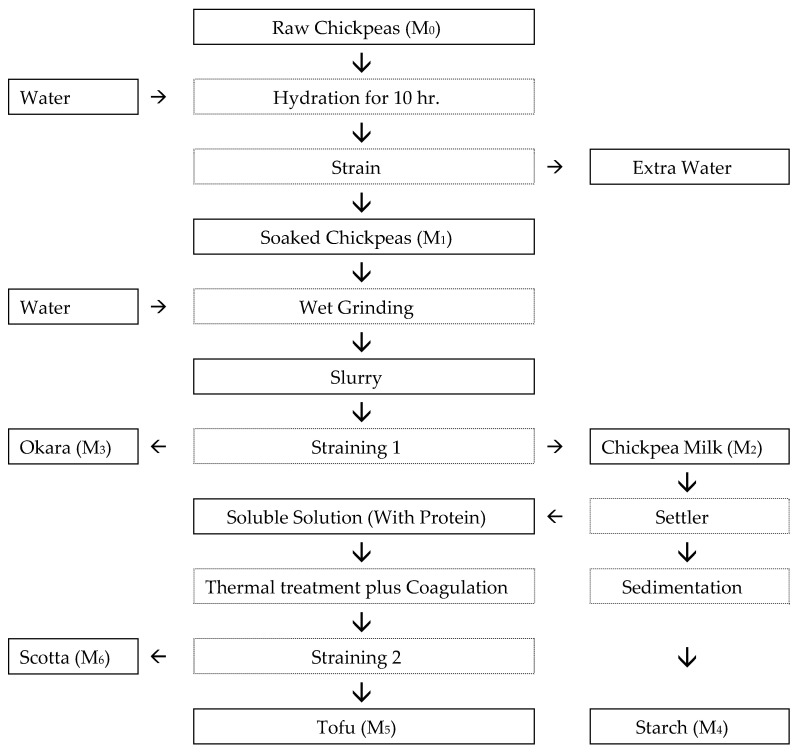
Mass balance flow chart of chickpea tofu production. All streams denoted M_0_–M_6_ represent measured mass values (wet basis) at key unit operations.

The process flow for tofu production and fractionation is summarized in Figure 1, showing key mass streams tracked for yield and component recovery analysis. 

### 3.2. Physiochemical Properties

All values are averages of five independent extractions (n = 5). For each extraction, moisture, crude fat, crude protein, crude fiber, and ash were analyzed in triplicate. All values, except for moisture, were expressed on a dry-weight basis.

Moisture content was determined using the AOAC official method 930.15 [37,38]. Samples were dried in a hot air oven at 135 °C for 2 h. The moisture content was calculated from the weight loss after drying.

Fat content was determined by ether extraction according to AOCS official method Am 5-04 [39].

Protein was determined by the Kjeldahl method according to AOAC 954.01 [40], with a conversion factor of 6.25 (N × 6.25) [41,42]. Protein content was calculated using the standard 6.25 factor to allow comparison with soy tofu studies conducted by Cai & Chang, 1997 [6] and Cai et al., 2002 [7], while recognizing that legume-specific factors (~5.7) may yield slightly lower values [3,34].

Crude fiber content was determined using an ANKOM 200 Fiber Analyzer (ANKOM Technology, Fairport, NY, USA) following acid–base digestion per AOAC 962.09 [43,44]. This method determines crude fiber, defined as the organic residue remaining after sequential digestion with 0.255 N sulfuric acid and 0.313 N sodium hydroxide.

The ash content was determined by ashing samples at 550 °C to constant weight (4–6 h) [42,45].

Carbohydrate content was calculated by difference and is reported as the net carbohydrate (Equation (1)).

Net Carbohydrate (%) = (100 − %Moisture − %Protein − %Fat − %Ash − %Crude Fiber)(1)

### 3.3. Calculated Attributes

#### 3.3.1. Conversion of Chickpeas to Okara, Starch, and Tofu

All masses, including wet masses of okara (M_3_), starch (M_4_), and tofu (M_5_), were measured gravimetrically using an analytical balance (±0.01 g) (Figure 1). The resulting average dry matter content for each stream was applied to single gravimetric yield measurements in all subsequent runs.

Mass yields of okara, starch, and tofu were expressed on a wet and dry basis. Wet basis yields (kg product∙kg^−1^ raw chickpeas) were calculated following Equation (2):

Yield_wet,i_ = M_i_/M_0_ (2)

where

- M_0_ is the wet mass of raw chickpeas (kg);
- M_i_ is the wet mass of product i (kg; okara i = 3, starch i = 4, or tofu i = 5) (Figure 1).

Dry basis yields (kg dry product∙kg^−1^ raw chickpeas) were calculated following Equation (3):

Yield_dry,i_ = [M_i_ × DM_i_]/[M_0_] (3)

where

- M_i_ is the wet mass of product i (kg; okara i = 3, starch i = 4, or tofu i = 5) (Figure 1);
- M_0_ is the wet mass of raw chickpeas (kg) (Figure 1);
- DM_i_ is the dry matter of product i (kg; okara i = 3, starch i = 4, or tofu i = 5).

#### 3.3.2. Operating Energy Needed

The thermal energy input required to heat chickpea milk from an initial temperature of 0 °C to boiling (100 °C) was calculated by considering only sensible heat, assuming negligible evaporation during the 5 min boil in a closed vessel. To improve accuracy, the specific heat capacity Cp of chickpea milk at 8 °Bx was estimated as 3.8 kJ·kg^−1^·K^−1^, based on published values for soymilk with similar solids content [46]. The theoretical energy requirement was calculated using Equation (4):

Q_B_calculated_ = M·Cp·ΔT (4)

where

- Q_B___calculated_ is the sensible heat required for boiling (kJ);
- M is the mass of chickpea milk (kg);
- Cp is the specific heat capacity (3.8 kJ·kg^−1^·K^−1^) [46];
- ΔT is the temperature change (100 K).

The mass of chickpea milk used in the boiling energy calculation corresponded to the amount required to produce 1 kg of fresh tofu with 70% moisture, based on the measured yield after starch sedimentation and coagulation. Calculations assumed an initial chickpea milk temperature of 0 °C, following the standard industrial practice of conservative baseline modeling [47], and heat losses to the environment were excluded to simplify the comparative analysis.

The energy required to dry the products was calculated using the latent heat of vaporization of water (Equation (5)):

Q_D_calculated_ = M_v_·H_latent_ (5)

where

- Q_D_calculated_ is the thermal energy needed for drying (kJ);
- M_v_ is the mass of water to be evaporated (kg);
- H_latent_ is the latent heat of water (2260 kJ/kg).

The amount of evaporated water was determined from the difference between the initial mass of each fresh product (tofu, okara, scotta, starch) and its corresponding final dry weight. For the purpose of calculating energy requirements across product streams, the following approach was adopted:

- Tofu powder: derived from fresh tofu containing 70% moisture.
- Okara powder: derived from wet okara with 70% moisture, collected during the straining step.
- Starch powder: derived from wet starch sediment containing 55% moisture, following the 1.5 h sedimentation phase.
- Scotta powder: derived from liquid scotta with a soluble solids content of 3 °Bx.

All calculated energy values were converted to kilowatt-hours (kWh) using the factor: 1 kWh = 3600 kJ. These values represent theoretical minimum energy requirements under idealized conditions, assuming no thermal losses or equipment inefficiencies.

### 3.4. Fresh Basis Mass Balance of Streams

To standardize process reporting, all material flows were normalized to an input of 1.0 kg of raw chickpeas (13.45% moisture) hydrated with 3.0 kg of water. The fresh basis wet mass of each stream was recorded or inferred, and moisture contents were determined, as described in Section 3.2. Values are reported as mean ± standard error from five independent trials (n = 5). The resulting mass flows are summarized in Table 2.

## 4. Results

All compositional values reported in this section are expressed on a dry basis, unless explicitly stated otherwise.

### 4.1. Raw Chickpea Nutritional Results

The proximate composition of raw chickpeas used in this study is presented in Table 3. All nutrients except moisture are expressed as a dry matter basis. Total carbohydrates were estimated by difference, while digestible carbohydrates were calculated by subtracting the crude fiber from total carbohydrates. It represents the mean ± standard error based on five replicates (n = 5).

According to the Codex standard for certain pulses (CXS 171-1989), the maximum moisture content for chickpeas ranges between 14% and 16%, depending on climate and storage conditions [48]. The moisture content found in this study (13.45%) falls below this limit, indicating good storage stability.

A comprehensive review by Hall et al. (2017) [2], supported by other authors such as Jukanti et al. (2012) [10], Kaur and Prasad (2021) [49], and Rachwa-Rosiak et al. (2015) [50], reports chickpea protein content ranging between 17 and 23%, fat around 5–6%, and fiber around 8–10%, aligning well with the current findings. The results (Table 3) fall within these established nutritional ranges, reinforcing the compositional adequacy of the chickpea cultivar used as a suitable raw material for high-protein tofu production.

The compositional profile of the raw chickpeas used in this study confirms their nutritional adequacy as a protein-rich legume for further processing. The combination of 20% protein, low fat, and appreciable fiber confirms the suitability of these chickpeas for tofu-style product development. The low moisture content also complies with Codex standards, ensuring safe storage conditions. These characteristics underpin the techno-economic and nutritional rationale for valorizing chickpeas into high-protein tofu, especially in the context of sustainable and local plant-based protein innovations.

### 4.2. Okara, Starch, and Tofu Nutritional Results

The proximate composition of the three major fractions (okara, starch sediment, and tofu) obtained from the chickpea-based tofu process is presented in Table 4. Each fraction exhibited a distinct nutritional profile that reflects its functional role in the separation process.

The okara fraction, composed primarily of insoluble dietary components, was notably high in fiber (10.27% ± 0.39 %) and retained moderate amounts of protein (8.54% ± 0.40%) and fat (3.15% ± 0.22%). Its high carbohydrate content was largely due to residual starch.

The starch sediment exhibited a very high total carbohydrate content (95.11% ± 0.06%), with minimal protein, fat, ash, and fiber, indicating an efficient separation of low-protein starch from the chickpea milk.

The tofu was markedly enriched in protein (56.18% ± 1.38%), with elevated fat (12.21% ± 0.59%) and ash (6.29% ± 1.43%) contents. Its low fiber content (1.55% ± 0.22%) confirms the effectiveness of protein and fat concentration within the curd matrix.

Together, these data demonstrate clear compositional partitioning across all four outputs and reinforce the technical feasibility of nutrient stream separation through this chickpea-based tofu process.

### 4.3. Mass Balance of the Process

A fresh basis mass balance normalized to 1.0 kg raw chickpeas (13.45% moisture) plus 3.0 kg soak water is summarized in Table 2. Across five trials, soaking increased seed mass to 1.929 kg ± 0.032 kg. After filtration and a 1.5 h settling step, the decanted milk used for coagulation was 5.126 kg ± 0.335 kg, measured directly. Coagulation and drainage yielded 0.742 kg ± 0.021 kg of fresh tofu and 4.384 ± 0.334 kg of scotta, consistent with mass closure. The insoluble fractions were 1.032 kg ± 0.055 kg of wet okara and 2.273 kg ± 0.155 kg of starch sediment. These values provide transparent fresh basis accounting of each stream and align with expectations for curd–whey separations.

For comparability with the prior tofu yield literature [7,19,26], conversion ratios are also presented in Table 5. These values express the kilograms of raw chickpeas required to produce 1 kg of each product fraction and are intended as complementary indicators to the fresh basis flows shown in Table 2. Raw chickpeas with an initial moisture content of 13.45% ± 0.002% absorbed water during soaking, reaching a post-soaking moisture of 46.96% ± 0.016%. This corresponded to a soaking ratio of 1.93 kg ± 0.032 kg of soaked chickpeas per kilogram of dry chickpeas. When adjusted for the initial moisture of raw chickpeas (13.45%), the soaked mass was equivalent to 0.523 kg ± 0.009 kg per 1 kg of raw chickpeas on an as-received basis.

Among solid fractions, tofu required the least amount of raw chickpeas per kilogram of product, with a dry conversion ratio of 4.96 kg ± 0.15 kg. This indicates a high dry matter recovery from the chickpea milk. Okara and starch were recovered in appreciable quantities, reflecting the high fiber and carbohydrate content of chickpeas. The coagulation whey (scotta) was obtained at a conversion ratio of 0.289 kg ± 0.038 kg, confirming that approximately 4.35 kg of wet whey is recovered per kg of raw chickpeas. Its dry matter recovery was lower but still notable at 9.649 kg ± 1.277 kg chickpeas per kg whey powder.

These results support the feasibility of valorizing all major fractions, confirming the efficiency of a chickpea-based tofu system with minimal residual loss.

#### Component Recovery and Partitioning

To assess nutrient distribution across the chickpea-based tofu production process, a component recovery analysis was performed. The recovery of protein, fat, ash, fiber, and carbohydrates was calculated for okara, starch, and tofu, based on their respective yields and compositions. Values represent the percentage of each nutrient originally present in 1 kg of raw chickpeas (dry basis) (Table 6).

Protein was primarily recovered in the tofu fraction (59.09% ± 2.90%), consistent with efficient curd formation and protein coagulation. Okara retained 13.45 % (±1.44%) of the original protein, while starch retained only 2.56% (±0.20%), confirming effective protein separation during the settling stage.

Fat showed a similar pattern: tofu accounted for the largest share (43.22% ± 3.22%), with notable retention in okara (16.67% ± 1.92%) and minor carryover into the starch layer (2.20% ± 0.27%). This supports the role of hydrophobic interactions and fat entrapment within the protein matrix during coagulation.

Fiber was markedly retained in okara (34.48% ± 0.75%), consistent with the early-stage separation of insoluble solids by cloth filtration. Smaller fiber contributions were detected in starch (1.30% ± 0.07%) and tofu (3.51% ± 0.49%).

Net carbohydrate recovery was highest in okara (40.28% ± 2.58%), followed by starch (21.59% ± 1.83%) and tofu (8.41% ± 0.54%). This distribution highlights the successful fractionation of chickpea solids: starch captured a purified carbohydrate stream, okara retained a mix of fiber and residual sugars, and tofu was selectively enriched in protein and fat.

Ash recovery was not calculated due to the addition of calcium chloride during the coagulation step, which introduced exogenous minerals and distorted native ash levels. Similarly, total carbohydrate was not separately reported since it includes fiber, which was already analyzed as an independent component. For this reason, only net carbohydrate recovery was assessed to reflect true digestible carbohydrate partitioning.

The summed recoveries across the three fractions reached 75.09 % (±3.13%) for protein, 90.37% (±5.16%) for fat, and 77.53% (±4.48%) for net carbohydrates. The remaining 25% discrepancy for protein and 23% for net carbohydrates can be attributed to soluble proteins and carbohydrates in the scotta fraction, which were not quantified but are consistent with reports of protein migration into tofu whey. Furthermore, recovery of fiber (39.3%) was comparatively lower. This is likely attributable to physical losses during processing, including adhesion of fine fiber particles to the straining cloth.

In summary, the process successfully partitioned chickpea macronutrients into distinct functionally relevant streams: tofu concentrated protein and fat, okara retained fiber and residual carbohydrates, and starch served as a purified carbohydrate source. These results demonstrate the potential for a low-waste chickpea bio-refinery model with multiple valorization pathways.

### 4.4. Estimated Energy

The theoretical minimum energy required to produce 1 kg of each product stream was estimated based on boiling and drying energy calculations. The values assume ideal conditions with no thermal losses and are summarized in Table 7.

Fresh tofu (70% moisture) required 0.798 kWh/kg, corresponding to the energy input needed to boil the chickpea milk before coagulation. Tofu powder production, which includes both boiling and drying stages, required a substantially higher energy input of 4.109 kWh/kg.

Byproduct streams required only drying energy, as the boiling step was attributed exclusively to tofu. Okara powder required 1.465 kWh/kg, reflecting moderate water content at the time of recovery. Starch powder was more energy-efficient, requiring 0.767 kWh/kg, due to its lower initial moisture content following sedimentation.

In contrast, scotta powder exhibited the highest energy demand at 20.298 kWh/kg. This elevated value is attributable to the very low solids concentration (~3 °Bx) of the liquid whey, which necessitates the evaporation of large quantities of water per unit of dry matter.

These values provide a comparative reference for evaluating the energy efficiency of each product pathway and highlight the trade-offs associated with valorizing dilute liquid streams like scotta.

## 5. Discussion

### 5.1. Nutritional Transformation

The chickpea-based tofu system developed in this study effectively transformed seed macronutrients into a high-protein curd, while producing clearly partitioned coproducts in the form of okara and purified starch. Protein content in the tofu curd reached 56.2% on a dry basis, nearly triple the level found in raw chickpeas (~20.5%). This concentration is comparable or slightly higher than the typical soy tofu benchmarks (40–50% d.b.), even under comparable coagulation and pressing conditions that yield similar curd firmness [7,26]. This increase can be attributed to the high efficiency of the CaCl_2_ coagulant and a 1.5 h starch-reducing settling stage, which minimized dilution and enhanced curd compaction.

In addition to its favorable protein concentration, chickpea tofu offers important nutritional advantages over soy-based counterparts. Notably, chickpea proteins exhibit lower levels of antinutritional factors, particularly trypsin inhibitors, which are present in soy at levels that often require thermal inactivation [6,46]. This enhances the digestibility of chickpea tofu and may reduce gastrointestinal discomfort in sensitive individuals. Furthermore, chickpea tofu is naturally free from major soy allergens, including Gly m 5 (β-conglycinin) and Gly m 6 (glycinin), which are responsible for the majority of immunoglobulin E (IgE)-mediated soy allergies [34]. This absence positions chickpea tofu as a hypoallergenic alternative for populations with soy intolerance or atopic risk, particularly in pediatric, geriatric, or clinical nutrition contexts.

In terms of macronutrient retention (Table 6), 75.1% of the initial protein was recovered across the tofu, okara, and starch streams, with 59.1% retained in the tofu fraction alone. These values are consistent with, or slightly higher than, those reported for soy tofu systems utilizing calcium chloride (CaCl_2_) or calcium sulfate (CaSO_4_) as coagulants [6,29]. Although chickpeas contain less protein than soybeans on a dry weight basis (~20.5% vs. ~36–40%), the final chickpea tofu product remains nutritionally comparable. This is primarily due to the high protein retention efficiency observed during chickpea tofu production. Specifically, 59.1% of the seed protein was retained in the tofu curd, exceeding the typical 35–45% retention reported for soy tofu prepared with calcium-based coagulants [6,29].

The higher partitioning efficiency effectively compensates for the lower initial protein content of chickpeas, enabling the production of a curd with protein levels comparable to, even exceeding, those of soy-based counterparts on a per-weight basis. These findings underscore the suitability of chickpea proteins for thermal and salt-induced gelation, and further support the potential of chickpea tofu as a nutritionally viable alternative to conventional soy tofu.

Fat retention followed a similar trend, with 43.2% of the original lipid content recovered in tofu, and 90.4% across all fractions. This aligns with soy tofu systems, where lipids are hydrophobically entrapped within the protein gel network [26,27].

The ash content of tofu was relatively high (6.3%), reflecting mineral uptake from the added calcium coagulant, in agreement with soy studies such as Chang and Liu (2012) [26] and Silsin et al. (2021) [19], who both observed elevated ash values in calcium-treated soy tofu. Ndatsu and Olekan (2012) [27] further confirmed that different coagulants can significantly influence the mineral profile of the final curd.

In contrast, fiber recovery in tofu was minimal (3.5%), with 34.5% of total fiber accumulating in okara, and 1.3% in the starch fraction. This low fiber retention in the curd is consistent with previous reports on both chickpea and soybean tofu systems [6,19]. The most plausible explanation lies in the coarse insoluble nature of legume fiber, which does not participate in curd gelation and is instead retained in the filter cake or intermediate sediment layer. This observation supports the visual and physical separation noted during the settling step, where denser colloidal particles formed a layer distinct from both curd and starch.

Regarding net carbohydrates, 77.5% of original seed carbohydrates was recovered, with 40.3% in okara, 21.6% in starch, and 8.4% in tofu. The remaining losses may stem from dissolved sugars and oligosaccharides in scotta or measurement uncertainty. Prior studies confirm that soy and chickpea whey retain significant amounts of raffinose, stachyose, and low-molecular carbohydrates [12,51], supporting this interpretation.

Overall, the nutrient recovery profile illustrates an efficient transformation of seed components into three functional fractions, with tofu acting as a protein- and fat-rich matrix, okara as the fiber concentrate, and starch as a purified carbohydrate stream. These findings confirm the technical feasibility and nutritional advantages of the chickpea tofu process and lay the foundation for the environmental and techno-economic analyses.

### 5.2. Yield Transformation

Interpreting yield requires both the fresh basis balance (Table 2) and the comparative conversion ratios (Table 5); the latter are provided for continuity with the tofu-yield literature. To assess the material efficiency of the chickpea-based tofu system, Table 5 presents conversion factors quantifying the quantity of raw chickpeas required to produce 1 kg of each product or byproduct. These values reflect not only compositional concentration but also the impact of water absorption, gelation behavior, and moisture removal at each process stage. Because powdered values are on a dry basis, they serve as standardized reference points. Fresh equivalents at any target moisture (e.g., 70%) are obtained by the conversion above, ensuring consistent benchmarking of yields, nutrient density, and energy across product formats while keeping fresh tofu as the primary outcome. Sensory attributes such as texture and taste should be addressed in future studies, while the potential applications of the dried fractions in value-added products warrant further exploration.

The production of wet tofu (70% moisture) required 1.49 kg of dry chickpeas per kg, consistent with soy tofu systems which typically require 1.2–1.6 kg depending on bean variety and coagulant used [6,26]. However, the dry equivalent tofu powder demanded nearly 5 kg/kg, reflecting both the moisture-rich nature of fresh tofu and the energy-intensive nature of drying, a well-documented challenge in legume-based protein gels [46].

For coproducts, okara powder required 3.26 kg of raw chickpeas per kg, whereas the wet counterpart (70% moisture) required only 1.03 kg/kg. This wide gap highlights okara’s high fiber and water-binding capacity, similar to soy okara which can retain up to 76% moisture post-filtration [19,29]. Such high moisture imposes considerable drying costs and necessitates low-temperature stabilization or fermentation strategies for valorization.

The starch powder conversion value (7.56 kg/kg) underscores the modest starch yield inherent in chickpeas, which contain approximately 40% carbohydrates but lower extractable starch than cereal grains [12]. The success of the 1.5 h settling step in isolating starch with high purity is notable, especially given that traditional soy tofu processes do not produce a separate starch fraction [6]. When used in semi-dry form (50% moisture), the yield efficiency improved to 2.27 kg/kg, offering an intermediate option for cost-conscious processors.

Scotta powder exhibited the highest conversion ratio at 9.65 kg/kg, revealing its dilute nature and the substantial energy cost of converting it into a storable format. However, tofu whey (scotta) is increasingly recognized as a source of bioactive compounds, including oligosaccharides, saponins, and isoflavones [51].

These yield patterns demonstrate the non-linear mass economy of pulse-based tofu systems, in which the primary protein curd is relatively efficient, but coproducts require careful consideration of moisture handling and market fit. The present data support a multi-stream processing strategy where tofu, okara, starch, and scotta are managed in an integrated manner. This framework enables more accurate techno-economic modeling, energy budgeting, and environmental impact assessment.

### 5.3. Techno-Economic Analysis

This section evaluates the economic feasibility of chickpea tofu and its associated coproducts under small-scale production conditions. It integrates empirical energy modeling, adjusted thermal efficiencies, and published cost benchmarks to assess product configurations and valorization options in low-resource or decentralized settings.

#### 5.3.1. Fresh Tofu: Input Cost and Efficiency

Chickpea tofu production offers a cost-effective approach for localized protein delivery. Under experimental conditions, 1.487 kg of raw chickpeas was required to produce 1 kg of fresh tofu (70% moisture). With chickpeas priced at USD 1.19/kg [52], and a boiling-stage thermal energy demand of 0.798 kWh/kg, the direct energy cost was USD 0.26, assuming an electricity rate of USD 0.33/kWh [53].

After applying a 30% overhead margin to cover coagulants, packaging, and equipment maintenance, the total cost of fresh tofu was estimated at USD 2.37/kg. This value aligns with cost structures reported for traditional soy tofu systems [6] and demonstrates affordability for smallholders, cooperatives, or community-based enterprises.

Notably, while soybeans have higher protein content than chickpeas, the protein retention in chickpea tofu was substantially higher (59.1%), resulting in a final product with comparable or superior nutritional density. This high conversion efficiency reinforces the economic value of chickpea tofu as a practical protein platform in regions lacking refrigeration or industrial infrastructure. A formal market-demand and logistics analysis was beyond the scope of this study and remains a priority for future work.

#### 5.3.2. Powdered Tofu: Shelf Stability and Cost Trade-Offs

Powdered tofu offers clear advantages in terms of shelf stability, portability, and suitability for institutional or humanitarian distribution, particularly in contexts where cold chain infrastructure is absent. Its lightweight dry format enables long-distance transport and long-term storage, making it an attractive option for school feeding programs, emergency rations, and food aid supply chains.

However, the production of powdered tofu is significantly more resource- and energy-intensive than that of fresh tofu. On average, 4.97 kg of raw chickpeas is required to produce 1 kg of tofu powder, reflecting both the moisture-rich nature of fresh tofu and the concentration effect required for a shelf-stable product. At 2023 market prices, this translates to a raw material cost of USD 5.91 per kilogram. In addition, modeled drying requirements of 4.109 kWh/kg add an estimated USD 1.36 per kilogram at prevailing electricity prices in Lebanon (USD 0.33/kWh). Together, these costs raise the minimum baseline to over USD 7.27/kg, excluding labor, packaging, and depreciation.

This cost profile limits the feasibility of powdered tofu for routine household consumption or informal markets. Instead, its value lies in specialized applications where extended storage and transport flexibility outweigh the higher production cost. Such contexts include humanitarian nutrition interventions, emergency preparedness, and institutional feeding programs. The elevated cost is largely attributable to the high moisture content of fresh tofu and the significant energy input required for dehydration, challenges that are particularly acute under high energy price conditions in Lebanon. Feasibility could be partially improved through coproduct valorization, as okara and starch require comparatively low drying energy and can contribute meaningfully to overall cost recovery.

#### 5.3.3. Coproduct Costing and Assumptions

The economic feasibility of tofu production can be enhanced through strategic valorization of its coproducts. The chickpea tofu process yields three distinct byproduct streams—okara, starch, and scotta—each with unique compositional and techno-economic characteristics.

Okara, rich in fiber and residual protein, represents the largest solid byproduct. It retains moderate moisture after pressing and can be dried or used fresh in feed, bakery, or fermented applications. With a modeled energy requirement of 1.465 kWh/kg and low processing complexity, okara flour production remains economically feasible under small-scale conditions, with a total cost estimated at USD 1.79/kg.

Starch is obtained through the settling step that separates suspended carbohydrates from the curd and fiber fractions. Although starch comprises a smaller proportion of the total mass, its low moisture content and simple composition make it an attractive target for drying or semi-dry usage. With an energy demand of just 0.767 kWh/kg, starch powder production was estimated at USD 1.42/kg, supporting its use in thickening agents or composite flours.

Scotta, the aqueous whey remaining after tofu pressing, presents the greatest processing challenge. With a solids content of only ~3 °Bx, drying requires over 20 kWh/kg of dry matter, translating to a prohibitive cost of USD 6.70/kg under current energy pricing in Lebanon. As such, drying is not considered viable in decentralized or artisanal systems. Instead, alternative uses, such as fermentation into lactic acid or beverages, incorporation into soups or porridges, or integration with okara for animal feed, are recommended.

#### 5.3.4. Summary Perspective

From a techno-economic perspective, fresh tofu remains the most efficient and accessible configuration for chickpea-based protein production. It requires modest inputs, minimal processing infrastructure, and delivers a nutritionally dense product at approximately USD 2.37/kg, making it highly suitable for smallholders, cooperatives, and decentralized food systems.

Powdered tofu, while valuable for targeted applications such as humanitarian aid or school feeding, incurs significantly higher production costs (>USD 7.27/kg) due to raw material requirements and the energy intensity of drying. Coproduct valorization—particularly okara and starch—can improve overall cost recovery, while scotta is better suited to non-thermal uses.

This tiered model—fresh tofu for local consumption, powdered tofu for strategic distribution, and coproducts for secondary markets—offers flexibility and resilience in energy-constrained or infrastructure-limited contexts, such as Lebanon and other semi-arid regions where plant-based proteins are increasingly relevant to food security and sustainable development. A formal market-demand and logistics analysis was beyond the scope of this study.

### 5.4. Valorization of Scotta: Drying Feasibility and Alternatives

Scotta, the aqueous fraction remaining after tofu pressing, presents the most significant techno-economic challenge among the process streams. With a soluble solids content of approximately three degrees Brix, its conversion into powder form requires the removal of more than 30 kg of water per kilogram of dry matter. Based on modeled energy demand, this would require over 20 kilowatt-hours per kilogram, resulting in a drying cost of approximately USD 6.70 per kilogram under prevailing electricity prices in Lebanon. These figures make scotta drying economically unjustifiable for small-scale or decentralized production.

Given these constraints, the valorization of scotta should focus on non-thermal strategies that preserve its nutritional potential without incurring additional energy burdens. Like soy whey, chickpea-based scotta contains soluble carbohydrates, minerals, peptides, and low-molecular-weight compounds that may support functional, nutritional, or microbial applications.

Feasible valorization options include the following:

- Lactic acid fermentation using existing pulse or dairy fermentation cultures, which can yield probiotic beverages or bio-acid solutions as mentioned by Peñas et al. (2006) [54].
- Direct culinary use as a base for soups, porridges, or fortified drinks, especially in institutional feeding settings.
- Microbial conversion for the production of bioactives or micronutrients such as vitamin B12, leveraging known capabilities of Propionibacterium species demonstrated by Yu et al. (2015) [55].
- Composting or animal feed blending, particularly in combination with okara to enhance nutrient recovery in agricultural or livestock systems.

These approaches support circular processing, reduce waste, and enhance the overall sustainability of chickpea tofu production. In contexts like Lebanon, where energy costs are high and infrastructure is limited, such strategies provide a practical path forward and complement the economic model built around tofu, okara, and starch.

### 5.5. Environmental Perspective (Conceptual)

No formal life-cycle assessment was conducted. The following discussion is conceptual and based on process simplification, mass balance, and energy indicators. Environmental performance is a central consideration in evaluating the feasibility and sustainability of chickpea tofu as a locally adaptable protein source. The process developed here makes use of whole chickpeas with high material efficiency and minimal waste. Key coproducts such as okara and starch can be valorized for food or feed applications, contributing to circular resource use in low-infrastructure settings.

Thermal energy inputs remain relatively modest, limited to boiling and dehydration. In comparison with conventional soy or dairy protein production, which often involves pressure cooking, enzymatic treatments, or centrifugation, the chickpea tofu process avoids high-intensity operations and complex post-processing. This simplification supports both energy savings and feasibility in decentralized environments.

Energy modeling confirms that low-intensity drying scenarios such as convection drying are more relevant for household-scale or rural operations, even if they are less efficient in terms of heat transfer. While not ideal for rapid throughput, they offer compatibility with existing infrastructure and may be adapted to renewable energy inputs.

The process also requires limited water, with no extensive washing, oil extraction, or neutralization steps. Emissions are minimal, since the process avoids combustion and uses no chemical solvents. Compared with published LCA studies, chickpea tofu processing exhibits lower direct energy demand per kilogram protein than soy tofu [56] and dairy curds [57], though further system-level assessment is needed to validate these comparisons. Chickpeas can be locally sourced in dryland regions, making the system compatible with climate-resilient cropping systems.

When viewed together with its high protein retention, efficient yield, and the potential for coproduct utilization, this chickpea-based tofu process supports sustainable food production and dietary diversification, particularly in semi-arid and resource-constrained regions.

### 5.6. Food Security Relevance (Perspective)

These considerations are framed as perspectives based on nutritional profile, equipment simplicity, and procurement frameworks rather than measured field outcomes. Chickpea-based tofu offers a practical and adaptable protein solution for food-insecure regions, combining simplicity, nutritional density, and compatibility with local agro-ecological conditions. Chickpeas are widely cultivated in arid and semi-arid regions, require limited agricultural inputs, and can be processed without industrial-scale infrastructure.

Importantly, chickpeas are not listed among the FAO/WHO priority food allergens, and severe allergic reactions are rare compared with soybeans or peanuts. This is significant in humanitarian contexts where peanut-based supplements have been widely used, as in WFP and UNICEF programs, but carry a higher risk of allergenic reactions. The U.S. Food and Drug Administration’s GRAS assessment for chickpea protein confirmed the absence of clinically significant cross-reactivity in peanut-allergic individuals [8]. This low allergenicity makes chickpea tofu suitable for population-scale feeding interventions, including school meal programs, emergency food distribution, and refugee nutrition initiatives, where allergen control is a logistical and public health priority.

The production process requires only whole chickpeas, water, heat, and filtration, with no additives or cold chain logistics. Fresh tofu can be consumed immediately, while powdered tofu enables long-term storage and transport in humanitarian supply chains. Powdered forms are lightweight, shelf-stable, and rehydratable with boiling water, allowing rapid preparation of protein-rich meals in field settings.

All major coproducts—okara, starch, and whey—can be repurposed into animal feed, fortified flours, or porridges, maximizing resource efficiency and reducing waste. This integrated model contributes directly to sustainable development goals 2 (zero hunger), 3 (good health and well-being), 12 (responsible consumption and production), and 13 (climate action) by promoting resilient food systems, reducing reliance on allergenic or imported proteins, and fostering circular use of agricultural resources.

Implementation challenges include variability during scale-up, limited access to drying infrastructure, and the need for expanded sensory evaluation across cultural contexts. Addressing these barriers will require technical adaptation, local capacity building, and targeted investment in appropriate equipment, such as small-scale presses and energy-efficient dryers. Previous humanitarian programs, which have distributed low-cost tofu presses and heating units in Asia and Sub-Saharan Africa [6], provide a proven implementation model, as these devices can operate using wood, charcoal, propane, or solar heat.

In practical terms, the success of chickpea tofu in supplementation programs will also depend on how it is consumed. Fresh tofu can be distributed to schools, community kitchens, or refugee camps and incorporated into local dishes such as soups, stews, and porridges. Powdered tofu, by contrast, lends itself to fortification strategies, where it can be blended into cereals, baked goods, or therapeutic rations without significantly altering sensory properties. This dual pathway of direct cooking for immediate use and fortification for supplementation enhances the versatility of chickpea tofu and strengthens its relevance across both emergency response and long-term nutrition initiatives.

The deliberate use of a household blender in the process further emphasizes its relevance to domestic settings. By demonstrating that a two-phase five-minute blending cycle effectively eliminates coarse particles in the okara, we show that chickpea tofu can be reliably produced using basic household appliances. This ensures that the method is accessible for use in community kitchens, refugee camps, and rural households, without the need for industrial mixers. This aligns with previous findings by Kim and Goldsmith (2021) [28], who highlighted the role of low-cost Soy Kit presses in supporting decentralized tofu production.

This study did not evaluate sensory attributes (taste, texture, aroma) or shelf-life stability of chickpea tofu. While the present work focused on composition and techno-economics, future research should systematically quantify firmness, syneresis, microbiological safety, and consumer acceptance of chickpea tofu.

## 6. Conclusions

This study demonstrates that high-protein tofu can be produced from whole chickpeas through a simple low-input process suited to both artisanal and humanitarian contexts. The product achieved 59.1% protein retention from seed input, exceeding typical soy tofu benchmarks, while avoiding major allergens linked to soy and peanuts.

Fresh tofu can be produced at ~USD 2.37/kg using only basic equipment and locally available fuels, supporting its adoption in smallholder and cooperative systems. Powdered tofu, though costlier due to drying energy demands, offers value in contexts requiring shelf stability and long-distance distribution. Coproducts such as okara and starch provide low-cost valorization opportunities, while whey can be repurposed through non-thermal uses.

The process has modest thermal and water requirements, aligns with climate-resilient cropping, and supports circular economy principles. Its nutritional density, low allergenicity, and compatibility with existing tofu-making infrastructure indicate potential suitability for school feeding, emergency rations, and development programs, pending further validation of sensory, safety, and acceptance parameters.

With targeted investment in small-scale equipment, adaptation to local preferences, and integration into procurement frameworks, chickpea tofu can contribute directly to sustainable development goals 2, 3, 12, and 13, advancing resilient and equitable protein access.

## Figures and Tables

**Table 1:** Unit operations and key processing parameters for laboratory-scale production of chickpea tofu.

Step	Name of Operation	Procedural Details *	Reference
1	Raw material preparation	Chickpeas (*Cicer arietinum* L.) were purchased then rinsed with potable water.	
2	Soaking	Chickpeas were immersed in three parts water (1:3 *w*:*w*) at ambient temperature (~22 °C) for 10 h, to attain ~2:1 (soaked/dry) hydration.	[26]
3	Wet grinding/slurry preparation	A Braun Jug Blender JB3060WH (800 W, max speed 24,000 rpm; De’Longhi Braun Household GmbH, Neu-Isenburg, Germany) was used for homogenization in two phases. First phase: Soaked chickpeas were blended with water at a 1:2 (*w*/*v*) ratio until uniform. Second phase: Water was added to reach a final 1:4 (*w*/*v*) ratio, and blended until a homogeneous mixture was obtained (~5 min).	[26,36]
4	Filtration (okara removal)	The slurry was passed through a double-layer muslin cloth; retained fiber (okara) was removed.	[26]
5	Starch sedimentation and milk decanting	The filtrate was held for 1.5 h; settled starch remained in the vessel while the clarified upper layer was decanted for curding. Starch sedimentation was achieved by manual decanting at a controlled ambient temperature (22–24 °C) without agitation	[7]
6	Thermal treatment	The decanted milk was heated to 98–100 °C for 3–5 min to denature proteins and inactivate enzymes.	[26]
7	Coagulation (CaCl_2_)	Hot milk (≈85 °C) treated with anhydrous CaCl_2_ at the rate of 2.5 g L^−1^; gentle stirring for 10 s, then standing 10 min formed curd.	[26]
8	Molding and passive drainage	Curd was ladled into a cloth-lined perforated mold and allowed to drain by gravity without pressing.	[26]
9	Refrigerated holding	Blocks were kept at 4 °C for 12 h for whey drainage and cooling prior to further testing.	[6]

*: For clarity, all mass balance calculations were normalized to a starting input of 1.000 kg of raw chickpeas (13.45% moisture) hydrated with 3.000 kg of water. Subsequent streams are reported on a fresh basis (wet mass and moisture) with dry matter composition as measured.

**Table 2:** Fresh basis mass balance of chickpea tofu production per 1 kg raw chickpea (mean ± SE, n = 5).

Stage	Stream	Mean ± SE
Input	Raw chickpeas	1
	Soak water *	3
Intermediate output **	Soaked chickpeas	1.98 ± 0.032
Intermediate streams	Okara (fresh)	1.032 ± 0.055
	Decanted milk (pre-coagulation)	5.126 ± 0.335
	Starch sediment (fresh)	2.272 ± 0.155
Final output	Tofu (fresh)	0.744 ± 0.021
	Scotta (whey)	4.384 ± 0.333

Stage-wise mass balances use measured unstandardized fresh masses. * Total water used for soaking; unabsorbed water was drained after soaking. ** The slurry, used as the input for the intermediate stream, was prepared by blending soaked chickpeas with water at a 1:4 (*v*:*v*) ratio.

**Table 3:** Proximate composition of raw chickpeas (mean ± standard error). All components except for moisture are expressed as a dry matter basis (d.b.).

Component	Values (Mean ± SE)
Moisture	13.45 ± 0.17
Protein (d.b.)	20.52 ± 0.76
Fat (d.b.)	6.03 ± 0.56
Ash (d.b.)	5.71 ± 0.98
Fiber (d.b.)	9.16 ± 0.14
Net Carbohydrates ^1^ (d.b.)	58.59 ± 1.24
Total Carbohydrates ^2^ (d.b.)	67.74 ± 1.18

^1^ Net carbohydrates: 100 − (% protein + % fat + % ash + % fiber) (dry basis). ^2^ Total carbohydrates: net carbohydrates% + fiber% (dry basis).

**Table 4:** Proximate composition of chickpea-derived okara, starch, and tofu (mean ± standard error, n = 5). All components except moisture are expressed as dry basis (d.b.).

Component	Okara (%)	Starch (%)	Tofu (%)
Moisture	71.42 ± 1.86	53.30 ± 2.18	68.90 ± 4.94
Protein (d.b.)	8.54 ± 0.40	3.77 ± 0.04	56.18 ± 1.38
Fat (d.b.)	3.15 ± 0.22	0.95 ± 0.05	12.21 ± 0.59
Ash (d.b.)	1.50 ± 0.05	0.18 ± 0.01	6.29 ± 1.43
Fiber (d.b.)	10.27 ± 0.39	0.89 ± 0.03	1.55 ± 0.22
Net Carbohydrates ^1^ (d.b.)	76.54 ± 0.31	94.22 ± 0.06	21.27 ± 2.38
Total Carbohydrates ^2^ (d.b.)	86.81 ± 0.64	95.11 ± 0.06	22.82 ± 2.37

^1^ Net carbohydrates: 100 − (% protein + % fat + % ash + % fiber) (dry basis). ^2^ Total carbohydrates: net carbohydrates% + fiber% (dry basis).

**Table 5:** Raw chickpeas required per 1 kg of product (kg/kg) (mean ± standard error).

Product	Conversion Value (kg Raw Chickpeas/kg Product)
Soaked chickpeas	0.523 ± 0.009
Okara powder	3.26 ± 0.154
Wet okara (70% moisture equivalent)	1.03 ± 0.515
Starch powder	7.575 ± 0.518
Wet starch (50% moisture equivalent)	2.273 ± 0.155
Tofu powder	4.957 ± 0.152
Wet tofu (70% moisture equivalent)	1.487 ± 0.507
Scotta (3 °Bx)	0.289 ± 0.038
Scotta powder	9.649 ± 1.277

Note: Conversion ratios are expressed as kilograms of raw chickpeas required per kilogram of product (kg/kg).

**Table 6:** Percent recovery of macronutrients in each major fraction (mean ± standard error, n = 5).

Nutrient	Okara (%)	Starch (%)	Tofu (%)	Sum (%)
Protein	13.45 ± 1.44	2.56 ± 0.20	59.09 ± 2.90	75.09 ± 3.13
Fat	16.67 ± 1.92	2.20 ± 0.27	43.22 ± 3.22	90.37 ± 5.16
Fiber	34.48 ± 0.75	1.30 ± 0.07	3.51 ± 0.49	39.29 ± 0.74
Net Carbohydrate ^1^	40.28 ± 2.58	21.59 ± 1.83	8.41 ± 0.54	77.53 ± 4.48

Note: Recovery values are expressed as a percentage of the nutrient content in 1 kg raw chickpeas. ^1^ Net carbohydrates: 100 − (% protein + % fat + % ash + % fiber) (dry basis).

**Table 7:** Theoretical minimum energy required to produce 1 kg of each product.

Product	Energy Required (kWh/kg Product)
Tofu (70% moisture)	0.798
Tofu powder	4.109
Okara powder	1.465
Starch powder	0.767
Scotta powder	20.298

Note: Values represent theoretical minimum energy requirements based on boiling (for tofu) and/or drying (for powders), calculated under ideal conditions with no thermal losses.

## Data Availability

The original contributions presented in the study are included in the article, further inquiries can be directed to the corresponding author.
